# Calorie Restriction With Exercise Intervention Improves Inflammatory Response in Overweight and Obese Adults: A Systematic Review and Meta-Analysis

**DOI:** 10.3389/fphys.2021.754731

**Published:** 2021-11-15

**Authors:** Yubo Liu, Feng Hong, Veeranjaneya Reddy Lebaka, Arifullah Mohammed, Lei Ji, Yean Zhang, Mallikarjuna Korivi

**Affiliations:** ^1^Exercise and Metabolism Research Center, College of Physical Education and Health Sciences, Zhejiang Normal University, Jinhua, China; ^2^Key Laboratory of Intelligent Education Technology and Application of Zhejiang Province, Zhejiang Normal University, Jinhua, China; ^3^Department of Sports Operation and Management, Jinhua Polytechnic, Jinhua, China; ^4^Department of Microbiology, Yogi Vemana University, Kadapa, India; ^5^Faculty of Agro Based Industry, Universiti Malaysia Kelantan, Kota Bharu, Malaysia; ^6^School of Communication and Arts, Shanghai University of Sport, Shanghai, China

**Keywords:** physical exercise, diet, C-reacting protein, meta-regression, inflammation, obesity

## Abstract

**Background/Purpose:** In this systematic review and meta-analysis, we assessed the effects of exercise (EX) combined with calorie restriction (CR) intervention on inflammatory biomarkers, and correlations between biomarkers and participants’ characteristics were calculated in overweight and obese adults.

**Methods:** An article search was conducted through PubMed, Web of Science, EMBASE, the Cochrane database, Scopus, and Google Scholar to identify articles published up to April 2021. Studies that examined the effect of EX + CR intervention on inflammatory biomarkers, including C-reactive protein (CRP), interleukin-6 (IL-6), and tumor necrosis factor-alpha (TNF-α), and compared them with a CR trial in overweight and obese adults were included. We calculated the pooled effect by meta-analysis, identified the correlations (between inflammatory biomarkers and participants’ characteristics) through meta-regression, and explored the beneficial variable through subgroup analysis. The Cochrane risk of bias tool and Methodological Index for Non-randomized Studies were used to assess the risk of bias for the included trials.

**Results:** A total of 23 trials, including 1196 overweight and obese adults, were included in the meta-analysis. The pooled effect showed that EX + CR intervention significantly decreased CRP levels (*P* = 0.02), but had no effect on IL-6 (*P* = 0.62) and TNF-α (*P* = 0.11). Meta-regression analysis showed that the effect of EX + CR on CRP, IL-6, and TNF-α changes was correlated with lifestyle behavior of adults (Coef. = −0.380, *P* = 0.018; Coef. = −0.359, *P* = 0.031; Coef. = −0.424, *P* = 0.041, respectively), but not with age and BMI. The subgroup analysis results revealed that participants with sedentary lifestyle behavior did not respond to EX + CR intervention, as we found no changes in CRP, IL-6, and TNF-α concentrations (*P* = 0.84, *P* = 0.16, *P* = 0.92, respectively). However, EX + CR intervention significantly decreased CRP (*P* = 0.0003; SMD = −0.39; 95%CI: −0.60 to −0.18), IL-6 (*P* = 0.04; SMD = −0.21; 95%CI: −0.40 to −0.01) and TNF-α (*P* = 0.006; SMD = −0.40, 95%CI: −0.68 to −0.12) in adults without a sedentary lifestyle or with a normal lifestyle. Furthermore, the values between sedentary and normal lifestyle subgroups were statistically significant for CRP, IL-6, and TNF-α.

**Conclusion:** Our findings showed that combination EX + CR intervention effectively decreased CRP, IL-6, and TNF-α in overweight and obese adults with active lifestyles, but not with sedentary lifestyle behavior. We suggest that ‘lifestyle behavior’ is a considerable factor when designing new intervention programs for overweight or obese adults to improve their inflammatory response.

## Introduction

According to the latest reports from the World Health Organization (WHO), worldwide prevalence of obesity has tripled since 1975. More than 1.9 billion adults (18 years or older) were overweight (39%) in 2016. Of these, more than 650 million adults (13%) were obese (WHO, 2021). The energy imbalance between calories consumption and calories expenditure is the fundamental cause of being overweight and obese. Increased intake of energy-dense foods (fat and sugar) over a period of time, and increased physical inactivity due to increased sedentary behavior are the primary contributors of being overweight and obese (Blüher, 2019; WHO, 2021). Weight gain has been associated with subclinical inflammation, which is mainly attributed to the secretion of various pro-inflammatory biomarkers (Saltiel and Olefsky, 2017). The inflammation caused by overnutrition or obesity is characterized by the activation of various immune cells, which release pro-inflammatory cytokines, such as tumor necrosis-alpha (TNF-α), interleukin-6 (IL-6), and C-reactive protein (CRP). Studies have shown that increased production of these inflammatory biomarkers is closely associated with the prevalence of chronic diseases, such as type 2 diabetes (T2D), cardiovascular diseases (CVDs), chronic kidney disease, and cancer (Coussens and Werb, 2002; Hotamisligil, 2006). It is further stated that more than 50% of all deaths worldwide are directly or indirectly linked with the progression of inflammatory-related diseases (Furman et al., 2019).

Recent evidence identified a strong association between obesity and severity of the coronavirus disease-19 (COVID-19), the ongoing global pandemic (Albashir, 2020). The severity of COVID-19 is represented by an excessive production of IL-6 (Mehta et al., 2020), TNF-α (Mortaz et al., 2021), and CRP (Chen et al., 2020). The need of intensive care for obese COVID-19 patients was reportedly higher compared to patients with a normal weight (Lighter et al., 2020), and disease severity increased with body mass index (BMI) and existence of inflammatory diseases (Simonnet et al., 2020). A study demonstrated that CRP and BMI were significantly higher in critical COVID-19 patients. Among the non-survivors, 88.24% of patients reportedly had a higher BMI (>25 kg/m^2^) (Peng et al., 2020). Therefore, controlling the release of inflammatory biomarkers is crucial for prevention of chronic diseases and to enhance the treatment efficiently in COVID-19 patients. Since excessive calorie intake and adiposity cause systemic inflammation, calorie restriction (CR) without malnutrition is a potential strategy to treat the inflammation and inflammatory diseases (Kökten et al., 2021). On the other hand, any form of physical activity or exercise has been documented to promote weight loss and decrease chronic low-grade inflammation, which together can attenuate several complications caused by inflammation (Nimmo et al., 2013). There is an increasing interest on investigating the combined effect of exercise (EX) and CR on an inflammatory system (Tolkien et al., 2019;Zheng et al., 2019). CR intervention (4-week) has been shown to decrease bodyweight and fasting blood glucose and insulin levels of obese women. This beneficial effect of CR was accompanied by decreased systemic inflammation in obese women (Ott et al., 2017). A previous meta-analysis concluded that exercise can improve the inflammatory response in overweight and obese adults, in which aerobic exercise had a greater beneficial effect (Yu et al., 2017). Another recent meta-analysis on older adults demonstrated that exercise intervention decreased IL-6 and CRP levels, but not TNF-α. However, this study was unable to establish a dose-related response to exercise and chronic inflammation (Monteiro-Junior et al., 2018).

Owing to the independent positive effects of exercise and CR interventions on inflammatory biomarkers, researchers wondered whether a combination of EX plus CR would amplify the beneficial effects compared to CR alone. To address this, Fisher at al. studied the independent effect of energy restriction alone and also in combination with exercise on changes in inflammatory mediators in overweight premenopausal women. The results showed that weight loss was associated with a reduction of inflammatory mediators; however, combining exercise and energy restriction did not alter the response (Fisher et al., 2011). Contrarily, another study reported a greater reduction of subcutaneous fat (not intra-abdominal fat) with a combination of EX plus CR compared to CR alone in postmenopausal women (van Gemert et al., 2019). Another study on obese sedentary adults reported no significant additional benefit of exercise on inflammatory response in a CR plus exercise intervention group (Trussardi Fayh et al., 2013). Compared with CR alone, combination of EX plus CR intervention exerts only a minor influence on adipocytokines and inflammatory cytokines in postmenopausal women (Giannopoulou et al., 2005). A recent meta-analysis concluded that a combination of EX plus CR largely decreased the inflammatory cytokines and CRP than CR alone in overweight and obese adults (Khalafi et al., 2021).

These controversial results of research studies and meta-analyses may be due to the differences in participants’ characteristics and/or intervention protocol. From the perspective of participants’ characteristics, no meta-analysis has yet analyzed the effect of EX plus CR intervention on inflammatory biomarkers in overweight and obese adults. It is therefore necessary to pool the data from EX plus CR interventions and examine the influence of participant’s characteristics on improving the inflammatory cytokines. In this study, we conducted a systematic review and meta-analysis of trials and explored the effect of EX plus CR on the alterations of CRP, IL-6, and TNF-α in overweight and obese adults. We further examined the association between characteristics of participants (age, BMI, and lifestyle behavior) and inflammatory biomarkers to identify the influential and effective variable that decreases inflammation.

## Methods

### Search Strategy

A thorough article search was conducted primarily using PubMed, Web of Science, EMBASE, the Cochrane database, Scopus, and Google Scholar. Specific keywords, including “exercise” OR “physical activity” OR “training” OR “aerobic exercise” OR “resistance exercise” OR “strength training” AND “caloric restriction” OR “restricted diet” OR “low calorie diet” OR “weight loss” were independently used with “inflammation” OR “interleukin-6” OR “tumor necrosis factor-α” OR “C-reactive protein” to search for articles. Furthermore, a manual search from the reference list of the included articles was conducted to identify relevant articles. Studies published until April 2021 were systematically searched by a team of authors (YL, FH, and YZ), and the influence of calorie restriction with exercise on inflammatory biomarkers was analyzed in overweight and obese adults with different lifestyle behavior.

### Inclusion and Exclusion Criteria

**FIGURE 1:**
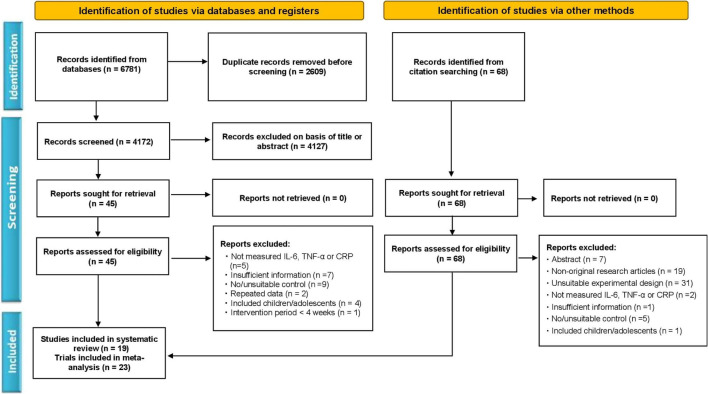
Flowchart of the study selection according to the Preferred Reporting Items for the Systematic Reviews and Meta-Analysis (PRISMA) method.

The article review and selection process was strictly followed according to the updated guidelines of Preferred Reporting Items for Systematic Reviews and Meta-Analysis (PRISMA) as described before (Moher et al., 2009; Page et al., 2021a, b). The detailed selection process with the number of articles in each step is depicted as a flowchart in Figure 1.

According to the selection criteria, a team of authors (YL, FH, and YZ) independently reviewed and assessed the relevant articles. Any differences in opinion among the authors on inclusion or exclusion of articles were solved by discussion with the corresponding author (MK). The inclusion criteria were: (1) All participants in the trials were overweight and obese adults; (2) the intervention group adopted calorie restriction with any type of therapeutic exercise (aerobic, resistance, mobility exercises, etc.), whilst the control group only underwent calorie restriction; (3) trials compared the effect of exercise and/or calorie restriction with respective controls; (4) trials reported inflammatory biomarkers (IL-6, TNF-α, or CRP) data as outcome measures after intervention, and (5) all studies were published in English as full-text articles. The exclusion criteria were: (1) Studies involving minors (<18 years) or animals; (2) articles with repeated data; (3) non-original research articles, such as protocols, case reports, meta-analyses, and systemic reviews, and (4) articles with inadequate information about the characteristics of the participants or intervention.

### Outcome Measures and Data Extraction

Two review authors, YL and FH, independently extracted the study characteristics, and the same was verified by another review author, MK. Three authors (VRL, AM, and LJ) provided in-depth analyses of the results and coordinated data extraction and interpretation. The information extracted from each study included values of inflammatory biomarkers (CRP, IL-6, and TNF-α), characteristics of participants (number, sex, baseline age, and baseline BMI), baseline lifestyle (sedentary or normal lifestyle), and details of intervention protocol (type of exercise, intensity, frequency, duration). Publication details, including name of authors, year of publication, and study conducted region were also extracted, and are described in Table 1.

**TABLE 1:** Characteristics of the included studies, presented in chronological order.

First author	Country	Sex	Sample EX + CR/CR	Participants characteristics			Exercise type/intensity	Frequency (t/wk)	Duration	Outcomes	MINORS
				Age (years)	BMI (kg/m2)	Life-style					
Cho et al., 2019	South Korea	M/F	9/8	EX + CR: 34.5 ± 5.7 CR: 33.5 ± 5.0	EX + CR: 28.0 ± 2.6 CR: 27.8 ± 3.4	NP	AE + RE: NR	≥3	8 weeks	CRP	–
Rejeski et al., 2019	United States	M/F	AE + CR: 79 RE + CR: 75 CR:68	66.9 ± 4.7	33.5 ± 3.5	NP	AE: 12–14RPE RE: 40–75%1RM	4	72 weeks	CRP, IL-6	–
Galedari et al., 2017	Iran	M	AE + CR: 12 RE + CR: 10 HT + CR: 10 CR: 8	AE + CR: 28.8 ± 6.1 RE + CR: 31.7 ± 7.7 HT + CR: 30.8 ± 7.6 CR: 32.6 ± 6.8	AE + CR: 28.9 ± 1.32 RE + CR: 29.0 ± 2.9 HT + CR: 29.6 ± 1.5 CR: 29.2 ± 2.4	NP	AE: 65–70%HR_max_ RE: 60–80%1RM HT: 90–95%HR_max_	3	12 weeks	TNF-α	–
Weiss et al., 2016	United States	M/F	19/17	EX + CR: 57 ± 7 CR: 57 ± 5	EX + CR: 28.3 ± 1.8 CR: 27.7 ± 1.7	SP	AE: moderate- and high-intensity	7	12–14 weeks	CRP	–
Lam et al., 2015	United States	M/F	8/8	EX + CR: 37.9 ± 1.8 CR: 39.0 ± 2.1	EX + CR: 27.9 ± 0.6 CR: 27.7 ± 0.5	NP	AE: NR	5	24 weeks	CRP, IL-6, TNF-α	–
Oh et al., 2014	Japan	M	52/20	EX + CR: 49.1 ± 1.3 CR: 53.2 ± 2.1	EX + CR: 29.2 ± 0.4 CR: 28.5 ± 0.8	NP	AE: >40%HR_max_	3	12 weeks	CRP, IL-6, TNF-α	18
Bouchonville et al., 2014	United States	M/F	28/26	EX + CR: 70 ± 4 CR: 70 ± 4	EX + CR: 37.2 ± 5.4 CR: 37.2 ± 4.5	SP	AE + RE AE: 65–85%HR_max_ RE: 65–85%1RM	3	48 weeks	CRP	–
Ryan et al., 2014	United States	F	37/40	EX + CR: 60 ± 1 CR: 61 ± 1	EX + CR: 32 ± 1 CR: 33 ± 1	SP	AE: 50–85%HRR	3	24 weeks	CRP	16
Lakhdar et al., 2013	Tunisia	F	10/10	EX + CR: 38.90 ± 4.37 CR: 38.90 ± 3.94	EX + CR: 32.98 ± 2.17 CR: 33.02 ± 1.89	NP	AE: 55–80%HR_max_	3	24 weeks	IL-6, TNF-α	–
García-Unciti et al., 2012	Spain	F	13/12	EX + CR: 48.6 ± 6.4 CR: 51.4 ± 5.5	EX + CR: 35 ± 3.1 CR: 34.6 ± 3.4	SP	RE: 50–80%1RM	2	16 weeks	IL-6	–
Fisher et al., 2011	United States	F	AE + CR: 43 RE + CR: 54 CR: 29	20–41	AE + CR: 28 ± 1 RE + CR: 28 ± 1 CR: 28 ± 1	SP	AE: 65–80%HR_max_ RE: 60–80%1RM	3	Until a BMI < 25kg/m^2^	CRP, IL-6, TNF-α	–
Snel et al., 2011	Netherlands	M/F	14/13	EX + CR: 56 ± 2 CR: 59 ± 2	EX + CR: 36.4 ± 1.1 CR: 37.9 ± 1.4	NP	AE: 70%VO_2max_	5	16 weeks	CRP, IL-6, TNF-α	18
Christiansen et al., 2010	Denmark	M/F	21/19	EX + CR: 37.5 ± 8 CR: 35.6 ± 7	EX + CR: 34.2 ± 3 CR: 35.3 ± 4	NP	AE: NR	3	12 weeks	IL-6	–
Straznicky et al., 2010	Australia	M	20/20	EX + CR: 54 ± 1 CR: 55 ± 1	EX + CR: 31.8 ± 0.8 CR: 32.2 ± 0.9	SP	AE: 65%HR_max_	3	12 weeks	CRP	–
Silverman et al., 2009	United States	F	46/40	EX + CR: 60 ± 5 CR: 58 ± 5	EX + CR: 32.1 ± 4.2 CR: 32.6 ± 4.6	SP	AE: 50–75%HRR	3	24 weeks	IL-6, TNF-α	19
Brochu et al., 2009	Canada	F	36/71	EX + CR: 57.2 ± 5.0 CR: 58.0 ± 4.7	EX + CR: 32.6 ± 4.9 CR: 32.2 ± 4.6	SP	RE: 65–80%1RM	3	24 weeks	CRP	–
Giannopoulou et al., 2005	United States	F	11/11	EX + CR: 57.4 ± 1.7 CR: 58.5 ± 1.7	EX + CR: 33.7 ± 1.9 CR: 34.3 ± 1.9	NP	AE: 65–70%VO_2max_	3–4	14 weeks	CRP, IL-6, TNF-α	–
Nicklas et al., 2004	United States	M/F	64/71	EX + CR: 68 ± 7 CR: 68 ± 5	EX + CR: 33.9 ± 5.6 CR: 34.4 ± 4.9	SP	AE + RE AE: 50–75%HRR RE: NR	3	72 weeks	CRP, IL-6, TNF-α	–
You et al., 2004	United States	F	17/17	EX + CR: 59 ± 1 CR: 57 ± 1	25–40	SP	AE: 50–70%HRR	3	24 weeks	CRP	–

*M, male; F, female; EX, exercise; CR, calorie restriction; AE, aerobic exercise; RE, resistance exercise; HT, high-intensity interval training; AE + RE, combination of aerobic and resistance exercise; SP, sedentary people; NP, normal people; NR, not reported; %HR_max_, percentage of maximal heart rate; %VO_2max_, percentage of maximal oxygen uptake; %HRR, percentage of heart rate reserve; %1RM, percentage of one-repetition maximum; RPE, ratings of perceived exertion (6–20); t/wk, times/week; MINORS, methodological index for non-randomized studies.*

### Quality Assessment

The quality of the included randomized controlled trials (RCTs) was assessed using the Cochrane risk of bias tool (Higgins et al., 2011). The Cochrane risk of bias assessment tool consists of seven domains, namely random sequence generation/allocation concealment (selection bias), blinding of participants/personnel (performance bias), blinding of outcome assessment (detection bias), incomplete outcome data (attrition bias), selective reporting (reporting bias), and other biases. The quality of each domain was rated as “low risk,” “high risk,” or “unclear,” and were indicated with green (+), red (−), and yellow (?) colors and symbols, respectively. The quality of trials was assessed by two authors (YL and FH), and discrepancies were resolved through discussion with a third reviewer (MK) to reach a consensus. The quality of the non-randomized controlled trials (non-RCTs) was assessed with the Methodological Index for Non-randomized Studies (MINORS) entry, and non-RCTs with MINORS scores > 12 were included in the study (Slim et al., 2003).

### Statistical Analysis

In this study, Cochrane Collaboration’s Review Manager (RevMan 5.3., Copenhagen, Denmark) was employed to analyze the effect of intervention (EX + CR) and participants characteristics on inflammatory biomarkers (IL-6, TNF-α, and CRP) of overweight and obese adults. The outcome measures were summarized through standardized mean difference (SMD) and 95% confidence intervals (95% CI). Inter-study heterogeneity was tested using *I*^2^ statistic, when *I*^2^ ≥ 50%, this means strong heterogeneity. Then meta-regression analysis followed by subgroup analysis were performed to assess the sources of heterogeneity, and to explore the effective characteristics that promote the benefits of intervention. We used STATA version 12 (StataCorp., College Station, TX, United States) to run the meta-regression analysis, and examined the relationships between effect size estimates and the following covariates: (1) baseline age, (2) baseline BMI, and (3) baseline lifestyle behavior. We found baseline lifestyle behavior (sedentary or normal lifestyle) was correlated with the changes of inflammatory mediators. Sedentary behavior refers to any walking behavior that is characterized by energy expenditure ≤1.5 metabolic equivalents (METs), while in a sitting, reclining, or lying posture (Tremblay et al., 2017; Thivel et al., 2018). The included trials were subgrouped based on the lifestyle behavior (sedentary or normal) of adults, and the effective lifestyle behavior in response to intervention was determined. We then assessed the differences between subgroups using the test for heterogeneity in Review Manager version 5.3. The Egger’s test was conducted to examine potential publication bias. The level of statistical significance was set at *P* < 0.05.

## Results

### Search Results and Selection of Studies

We initially obtained a total of 6,849 articles, including 6781 from the electronic databases (PubMed, Web of Science, Scopus, EMBASE, SportDiscus, ScienceDirect, and Google Scholar) search, and 68 from the manual search. After removing duplicates (2,677), the remaining 4,172 articles were carefully screened by title and abstract, and a further 4,116 were excluded because of unsuitability. Then the full text of the 56 articles was thoroughly reviewed, and 37 of them were excluded due to the following reasons; not measuring IL-6, TNF-α, or CRP levels, insufficient information, no/unsuitable control, repeated data, included children or adolescents, and intervention period less than 4 weeks. Finally, 19 articles consisting of 23 trials met the inclusion criteria, and were included in the systematic review and meta-analysis. The informative flowchart (PRISMA) of article search, screening, and selection is presented in Figure 1.

### Summary of the Included Studies

According to the inclusion and exclusion criteria, a total of 19 articles published between 2004 and 2019 were included for the systematic review, meta-analysis, and meta-regression analysis. These studies were from various parts of the world, most were from the United States (10), with one each from Australia, Canada, Denmark, Iran, Japan, Korea, Netherlands, Spain, and Tunisia. The total sample size was 1196 participants, consisting of 688 adults from the intervention group and 508 adults from the control group. The mean age of participants in the trials ranged from 18 to 75 years. The types of exercise intervention included aerobic (You et al., 2004; Giannopoulou et al., 2005; Silverman et al., 2009; Christiansen et al., 2010; Straznicky et al., 2010; Fisher et al., 2011; Snel et al., 2011; Lakhdar et al., 2013; Oh et al., 2014; Ryan et al., 2014; Lam et al., 2015; Weiss et al., 2016; Galedari et al., 2017; Rejeski et al., 2019), resistance (Brochu et al., 2009; Fisher et al., 2011; García-Unciti et al., 2012; Galedari et al., 2017; Rejeski et al., 2019), a combination of both aerobic and resistance (Nicklas et al., 2004; Bouchonville et al., 2014; Cho et al., 2019), and high-intensity interval training (Galedari et al., 2017). The exercise intervention duration ranged from 8 to 72 weeks with a frequency of 3 to 7 times per week. The characteristics of patients (sex, age, BMI, and life style behavior) and exercise intervention (type, intensity, frequency, and duration) are presented in Table 1.

### Exercise Plus Calorie Restriction Decreases C-Reactive Protein Levels in Overweight and Obese Adults

**FIGURE 2:**
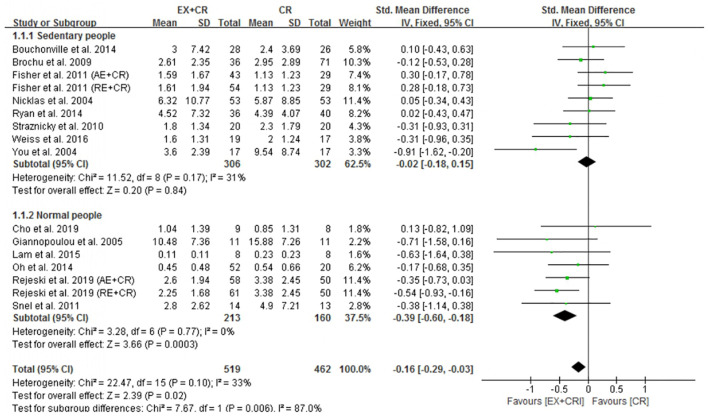
Forest plot of the effects of exercise plus calorie restriction (EX + CR) intervention on C-reactive protein (CRP) changes in overweight and obese adults with different (subgroup analysis) lifestyle behaviors.

Among the included trials (*n* = 23), 16 trials (981 participants) reported the effect of exercise plus CR intervention on alterations in CRP levels and compared the changes with a CR trial alone. The pooled outcome of meta-analysis revealed that exercise plus CR intervention significantly (*P* = 0.02) decreased CRP in overweight and obese adults. The standardized mean difference (SMD) of decreased CRP with EX + CR intervention was −0.16 with 95% CI: −0.29 to −0.03 (Figure 2 and Supplementary Figure 1). These findings revealed that overweight or obese adults required a combination of exercise and CR to decrease their CRP levels.

### Exercise Plus Calorie Restriction Does Not Affect IL-6 and TNF-α in Overweight and Obese Adults

**FIGURE 3:**
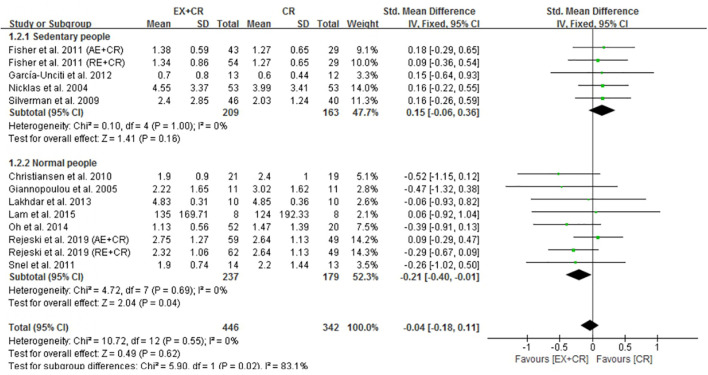
Forest plot of the effects of exercise plus calorie restriction (EX + CR) intervention on IL-6 changes in overweight and obese adults with different (subgroup analysis) lifestyle behaviors.

**FIGURE 4:**
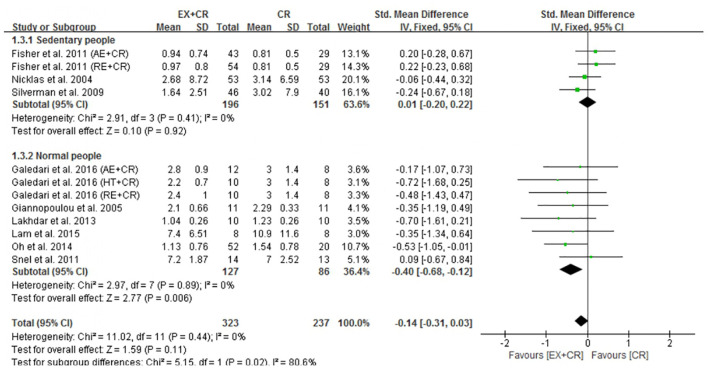
Forest plot of the effects of exercise plus calorie restriction (EX + CR) intervention on TNF-α changes in overweight and obese adults with different (subgroup analysis) lifestyle behaviors.

The changes in IL-6 concentrations with EX + CR intervention were reported in 13 trials, and the differences were compared with CR trials. Results of the pooled outcome showed that an EX + CR intervention for a period of 12–72 weeks did not influence IL-6 concentrations in overweight and obese adults (*P* = 0.62; SMD = −0.04; 95% CI: −0.18 to 0.11) (Figure 3 and Supplementary Figure 2). Besides, the effect of exercise plus CR on TNF-α concentration was evaluated in 12 trials of 560 overweight and obese adults. Similar to the IL-6 response, EX + CR-mediated changes in TNF-α were also not statistically significant (*P* = 0.11; SMD = −0.14; 95% CI: −0.31 to 0.03). Although TNF-α tended to decrease with EX + CR, such a change was not favorable to experimental intervention (Figure 4 and Supplementary Figure 3).

### Meta-Regression Analysis: Correlations Between Participants’ Characteristics and Inflammatory Biomarkers

Meta-regression analysis was conducted to identify the correlations between characteristics of participants (age, BMI, and lifestyle behavior) and inflammatory biomarkers (CRP, IL-6, and TNF-α). One of the key findings in our analysis is that ‘lifestyle behavior’ of participants was significantly correlated with the changes of CRP (Coef. = −0.380; *P* = 0.018), IL-6 (Coef. = −0.359; *P* = 0.031), and TNF-α (Coef. = −0.424; *P* = 0.041) after exercise plus CR intervention. In contrast, age and BMI of participants were not correlated with the changes of any inflammatory biomarkers (Table 2). These results indicate that lifestyle behavior, either sedentary or normal, is the key variable that is involved in improving the inflammatory mediators following EX + CR intervention.

**TABLE 2:** Meta-regression analysis for participants’ characteristics.

Inflammatory biomarkers	Characteristics of participants	Coefficient	Standard error	*T*-value	*P*-value
	Age	0.0026002	0.0099456	0.26	0.798
CRP	BMI	−0.0339752	0.0279858	−1.21	0.246
	Lifestyle	−0.3804664	0.142546	−2.67	0.018*
	Age	0.0104658	0.0082666	1.27	0.237
IL-6	BMI	−0.0206236	0.0304715	−0.68	0.513
	Lifestyle	−0.3592889	0.1457418	−2.47	0.031*
	Age	0.0125738	0.007944	1.58	0.152
TNF-α	BMI	−0.0121833	0.0379839	−0.32	0.755
	Lifestyle	−0.4249011	0.1811258	−2.35	0.041*

*Represents a significant correlation between the inflammatory biomarkers and characteristics of population.

### Subgroup Analysis Results

#### Normal Lifestyle With EX + CR Intervention Decreases C-Reactive Protein, Not Sedentary Lifestyle

Subgroup analysis was performed to identify the effective lifestyle (sedentary or normal) behavior of adults that could influence the beneficial effects of EX + CR intervention on inflammatory mediators. As shown in Figure 2, participants (213 from 7 trials) without sedentary behavior (normal lifestyle) showed greater beneficial effects of EX + CR intervention. This was evidenced by a substantial decrease of CRP levels (*P* = 0.0003; SMD = −0.39; 95%CI: −0.60 to −0.18) in normal-lifestyle adults after EX + CR intervention. However, CRP levels in participants with sedentary lifestyle behavior did not respond to EX + CR intervention. The heterogeneity of the subgroup analysis explained that the intervention effect of EX + CR on CRP levels was influenced by lifestyle behavior of adults (*I*^2^_in–subgroup_ < 50%; *I*^2^_between–subgroups_ = 87%) (Figure 2).

#### EX + CR Intervention Decreases IL-6 and TNF-α in Adults Without Sedentary Lifestyle

It is worth pointing out that EX + CR intervention had no effect on IL-6 in overweight and obese adults. Nevertheless, when trials separated participants based on lifestyle behavior, the IL-6 concentrations were significantly decreased in normal-lifestyle participants (237 from 8 trials) after EX + CR intervention (*P* = 0.04, SMD = −0.21; 95%CI: −0.40 to −0.01). Contrary to this, participants (209 from 5 trials) with sedentary lifestyle behavior did not show any positive response to EX + CR intervention (Figure 3). The heterogeneity (*I*^2^ = 83.1%) between the subgroups was strong, while the heterogeneity (*I*^2^ = 0%) within the subgroups was negligible, which reveals that the results of this study were relatively reliable (Figure 3).

Next, we found that a combination of exercise and CR intervention remarkably decreased TNF-α concentrations in participants with normal lifestyle behavior (*P* = 0.006; SMD = −0.40, 95%CI: −0.68 to −0.12), but not in adults with sedentary lifestyle (Figure 4). We further identified that there was no significant heterogeneity within the subgroups (*I*^2^ = 0%), but there was a significant heterogeneity between the subgroups (*I*^2^ = 80.6%), indicating the influential role of lifestyle behavior of participants on improving TNF-α (Figure 4).

### Summary of Risk of Bias

**FIGURE 5:**
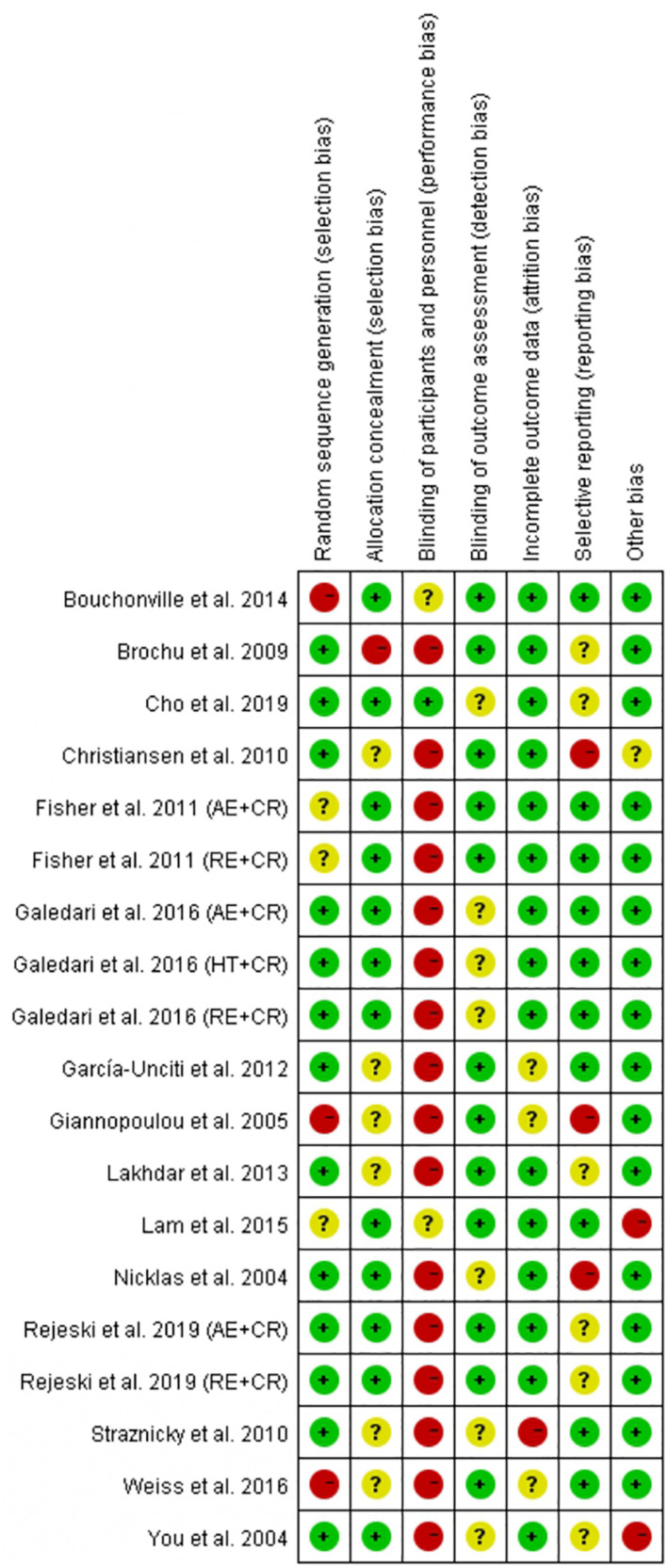
Summary of the risk of bias for the trials included in this meta-analysis.

Risk of bias in the included studies (19) was assessed according to the Cochrane risk of bias tool, and the judgment is presented in Figure 5. For the selection bias, more studies (13 trials) reported a low risk of random sequence generation, and 12 trials were judged to have a low risk of allocation concealment. Physical exercise is the main intervention among all the trials, and therefore it may not be feasible to adopt the blind method. As a result, several studies were judged as having a high risk of performance bias (16 trials). However, reporting of such a high risk of performance bias does not compromise the quality of the study (Hong et al., 2019; Liu et al., 2019). No studies were judged to have a detection bias. We identified one trial with an attrition bias, three trials with reporting bias, and two trials with other bias. The quality of the four non-RCT studies was evaluated using the MINORS evaluation criteria. One trial scored 19 points, two trials scored 18 points, and one trial scored 16 points.

## Discussion

To the best of our knowledge, this is the first systematic review and meta-analysis to compare the combination effect of exercise and CR with CR alone on inflammatory biomarkers in obese and overweight adults. We included 23 trials, which reported the changes in CRP, IL-6, and TNF-α concentrations with response to EX + CR and CR alone treatments. The pooled outcome results showed that combination of EX + CR intervention significantly decreased CRP levels in overweight and obese adults, while IL-6 and TNF-α did not respond to EX + CR intervention. Meta-regression analyses revealed that the changes in CRP, IL-6, and TNF-α were correlated with lifestyle behavior, but not correlated with age and BMI of participants. Subgroup analysis results further explored that normal lifestyle with EX + CR intervention effectively decreased CRP, IL-6, and TNF-α concentrations. However, baseline sedentary lifestyle behavior with EX + CR intervention could not decrease the inflammatory mediators in overweight and obese adults. These findings imply that participants’ lifestyle behavior, especially active lifestyle is important to achieve the beneficial effects of EX + CR intervention on improving the inflammatory biomarkers in overweight and obese adults.

IL-6 and TNF-α are the two important pro-inflammatory cytokines that mediate the process of inflammation and healing. However, excessive production of IL-6 and TNF-α over a period of time causes systemic inflammation, which is eventually involved in developing metabolic diseases, including CVDs, diabetes, various cancer types, chronic kidney disease, non-alcoholic fatty liver disease (NAFLD), and neurodegenerative conditions (Furman et al., 2019). Both cytokines, IL-6 and TNF-α, are responsible for the production and release of CRP, an acute phase protein that induces the inflammatory stage (Popko et al., 2010). Elevated levels of IL-6 and CRP are strongly associated with development of type 2 diabetes in healthy middle-aged women (Pradhan et al., 2001). Several lifestyle factors, including physical inactivity, poor diet, nighttime blue light exposure, smoking, and psychological stress are reported to promote systemic inflammation. Therefore, lifestyle modifications may reduce inflammatory proteins and improve metabolic health (Furman et al., 2019; Kökten et al., 2021). Studies have targeted these modifiable risk factors to decrease the inflammatory response, however, the corresponding reduction of inflammatory biomarkers is limited due to various reasons. Here, we addressed the influential role of lifestyle behavior on improving CRP, IL-6, and TNF-α with exercise plus CR intervention.

Exercise and dietary intake are important lifestyle interventions that are reported to orchestrate inflammatory biomarkers independently or together. A study with a very low calorie diet (CR) for 4 weeks has been shown to reduce inflammatory biomarkers in obese women, and this beneficial effect of CR was accompanied by a significant weight loss (Ott et al., 2017). In a randomized clinical trial, a modified alternate-day fasting diet was reported to be more effective than CR on weight loss and reduction of CRP levels in patients with metabolic syndrome. Despite this, the changes in IL-6 and TNF-α were not statistically different between the diets (Razavi et al., 2021). On the other hand, a meta-analysis reported positive effects of aerobic exercise intervention on reduction of CRP, IL-6, and TNF-α in middle-aged and older adults, but not on IL-4 (Zheng et al., 2019). The anti-inflammatory effect of exercise may be attributed to a decrease of adipose tissue; however, its independent effect on inflammation has not yet been elucidated. Evidence suggests that exercise can directly affect the immune cells by regulating systemic inflammatory mediators without relying on the loss of bodyweight (Gleeson et al., 2011). The anti-inflammatory effect of exercise is independent of weight loss and it can inhibit pro-inflammatory mediators, stimulate anti-inflammatory pathways, and thereby regulate insulin sensitivity. Nevertheless, it is not clear whether a combination of exercise and CR has a greater beneficial effect than that of CR alone on inflammatory cytokines in obese adults.

One of the key findings of our meta-analysis is that exercise plus CR has greater beneficial effects than CR alone in decreasing inflammatory mediators when participants are subgrouped based on their lifestyle behavior. Lifestyle modification with the Mediterranean diet (CR) and exercise intervention for 2 years induced significant weight loss and reduced CRP, IL-6, and TNF-α in healthy obese women. Nevertheless, the deteriorated adipokine profile was not improved with combination of CR and exercise intervention (Gomez-Huelgas et al., 2019). In a recent meta-analysis, exercise plus CR intervention significantly decreased IL-6 and TNF-α levels and marginally decreased CRP in overweight and obese adults (Khalafi et al., 2021). In contrast to our findings, subgroup analysis for BMI showed a significant decrease of CRP and TNF-α with higher BMI values, but not IL-6 (Khalafi et al., 2021). We performed meta-regression analysis for participants’ characteristics (age, BMI, and lifestyle), and found that lifestyle behavior is significantly correlated with decreased CRP, IL-6, and TNF-α after exercise plus CR intervention. BMI and age variables were not correlated with decreased inflammatory biomarkers in overweight and obese adults. Here, our findings emphasize the importance of lifestyle behavior in enhancing the beneficial effects of exercise plus CR intervention. The differences with previous findings may be due to the differences in population characteristics. Our study included only overweight or obese adults, and children below 18 years were excluded. Therefore, our conclusions are convincing to construct an intervention program for overweight or obese adults to improve their inflammatory response.

The mechanism of improving inflammatory markers by exercise intervention is not completely clear, which may be achieved by improving the hypoxia state of adipose tissue (Dumitriu et al., 2007), promoting the phenotype transformation of macrophages in adipose tissue (Kawanishi et al., 2010), and regulating the function of peripheral blood cells (Yeh et al., 2009). Although changes in body weight and composition are not necessarily factors that affect inflammatory markers, changes in body composition (muscle and fat content) caused by exercise may indirectly affect the inflammatory response of an individual (Beasley et al., 2009). A meta-analysis compared the effects of exercise and CR on body weight and composition, and found that CR has a larger impact on total body weight loss, but exercise has a superior effect in reducing visceral adipose tissue (VAT) (Verheggen et al., 2016), which is an important source of pro-inflammatory factors, such as IL-6 and TNF-α. In addition, skeletal muscle is the key source of inflammatory mediators involved in systemic inflammation. A meta-analysis of 13 articles showed that resistance exercise-induced increased muscle mass contributes to control of inflammatory biomarkers in older adults (Sardeli et al., 2018). The positive effect of exercise on muscle mass is not limited to resistance exercise or high-intensity exercise. In a study, overweight to obese adults were randomly assigned into diet-induced weight loss alone or diet-induced weight loss combined with exercise intervention groups for 4 months. The results showed that diet-induced weight loss (intentional CR) significantly decreased skeletal muscle mass in overweight to obese adults. Interestingly, moderate aerobic exercise combined with an intentional weight loss trial attenuated the loss of skeletal muscle mass (Chomentowski et al., 2009).

Although exercise and CR are important strategies to curb the level of inflammation, paying attention to the different characteristics of participants is the primary concern to achieve the goal. This study emphasizes that individual lifestyle behavior (sedentary or non-sedentary) is the key factor to achieve an intervention effect. Sedentary behavior refers to any walking behavior characterized by energy expenditure ≤ 1.5 metabolic equivalents (METs), while in a sitting, reclining, or lying posture (Tremblay et al., 2017; Thivel et al., 2018). To the best of our knowledge, this is the first meta-analysis and subgroup analysis to evaluate the combination effect of EX + CR on inflammatory mediators in overweight and obese adults with sedentary behavior. We found that exercise plus CR intervention could not improve the CRP, IL-6, and TNF-α response in overweight and obese adults with long-term sedentary behavior. An existing meta-analysis mainly revealed the combination effect of EX + CR on inflammatory mediators from the aspects of intervention duration, exercise type, and BMI, but did not disclose the influence of sedentary behavior of participants on the effectiveness of the intervention (Khalafi et al., 2021).

It is well known that sedentary lifestyle behavior is the main cause of obesity, and both obesity and sedentary behavior are strongly associated with developing inflammatory-related diseases (Park et al., 2020). Apart from the fact that obesity can cause chronic inflammation, sedentary behavior itself also contributes to chronic inflammation and inflammatory diseases in the course of life (Burini et al., 2020). Adults who have sedentary behavior for a long time may have impaired metabolic health and cannot benefit from exercise or other interventions. Previous studies have reported that long-term sedentary behavior can lead to inflammation of subcutaneous adipose tissue, and negative health consequences, including type 2 diabetes, obesity, hypertension, and CVDs (Grøntved and Hu, 2011; Biswas et al., 2015). A multi-ethnic cross-sectional study with a large sample has shown that sedentary behavior is associated with high levels of TNF-α and leptin and low adiponectin-to-leptin ratios. The degree of these associations does not vary with ethnic groups, and is independent of related co-variables (including moderate to high intensity exercise) (Allison et al., 2012). Moreover, exercise combined with nutrient intake or new exercise methods seems to improve the inflammatory response. A study on sedentary men showed that high intensity exercise plus honey intake significantly decreased IL-6 concentrations, but had no effect on other inflammatory mediators (Bakhtyar et al., 2018). Another study on sedentary middle-aged men reported that cycling training decreased only CRP levels, while small-sided games decreased both CRP and IL-6 concentrations and increased muscle mass (Mendham et al., 2014). Therefore, it is suggested that we should further investigate the effect of exercise combined with nutrient intake or a new exercise protocol on inflammatory factors.

### Limitations

The intervention duration of the included trials ranged between 8 and 72 weeks. Although the duration range appears to be wide, this may not influence the final outcome of our study. Meta-regression analysis results also showed no significant correlation between intervention duration and inflammatory mediators. In addition, our study did not provide the statistical evidence to demonstrate the influence of exercise type, intensity, or frequency on inflammatory biomarkers. We included trials that performed any type of exercise, and therefore it is inconclusive which exercise type has greater beneficial effects in combination with CR. Further studies with meta-regression analysis are necessary to identify the influential role of exercise variables on changes in inflammatory mediators in overweight and obese adults.

## Conclusion

Our findings demonstrated that a combination of exercise with calorie restriction could improve the CRP, IL-6, and TNF-α response in overweight and obese adults with normal lifestyle behavior, but not in adults with sedentary behavior. Therefore, lifestyle behavior is the key variable that influences the beneficial effects of exercise plus CR intervention on inflammatory biomarkers. Whenever designing interventional programs for obese or overweight adults, ‘lifestyle behavior’ should be considered as an important characteristic of adults to achieve the goal of intervention on the inflammatory system.

## Data Availability Statement

The original contributions presented in the study are included in the article/Supplementary Material, further inquiries can be directed to the corresponding authors.

## Author Contributions

YL, FH, and MK designed the study. YL, FH, and YZ performed the article search and screening. YL and FH performed statistical analyses and drafted the manuscript. VL, AM, and LJ assisted in interpretation of data and provided additional suggestions. YL, FH, YZ, and MK reviewed the full-text articles and extracted the data. YZ and MK revised and finalized the manuscript. All authors read and approved the submission.

## Conflict of Interest

The authors declare that the research was conducted in the absence of any commercial or financial relationships that could be construed as a potential conflict of interest.

## Publisher’s Note

All claims expressed in this article are solely those of the authors and do not necessarily represent those of their affiliated organizations, or those of the publisher, the editors and the reviewers. Any product that may be evaluated in this article, or claim that may be made by its manufacturer, is not guaranteed or endorsed by the publisher.
