# Supplementary material for: Calorie Restriction With Exercise Intervention Improves Inflammatory Response in Overweight and Obese Adults: A Systematic Review and Meta-Analysis
Source: Front Physiol. 2021 Nov 15;12:754731. doi: 10.3389/fphys.2021.754731 (PMC8634604; doi:10.3389/fphys.2021.754731)
Supplement: Supplementary file 1 [file Data_Sheet_1.docx]

**Calorie Restriction with Exercise** **Intervention Improves** **Inflammatory** **Response** **in** **Overweight and Obese** **Adults: A Systematic Review and** **Meta-Analysis**Yubo Liu, Feng Hong, Veeranjaneya Reddy Lebaka, Arifullah Mohammed, Lei Ji,

Yean Zhang and Mallikarjuna Korivi

**Supplementary Figures:**

**
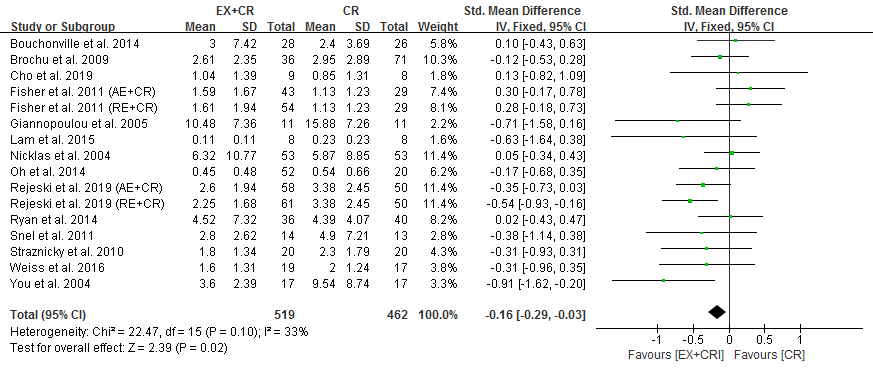
Figure 1S.** Forest plot of the effects of exercise plus calorie restriction (EX+CR) intervention and CR alone on CRP changes in overweight and obese adults.

**
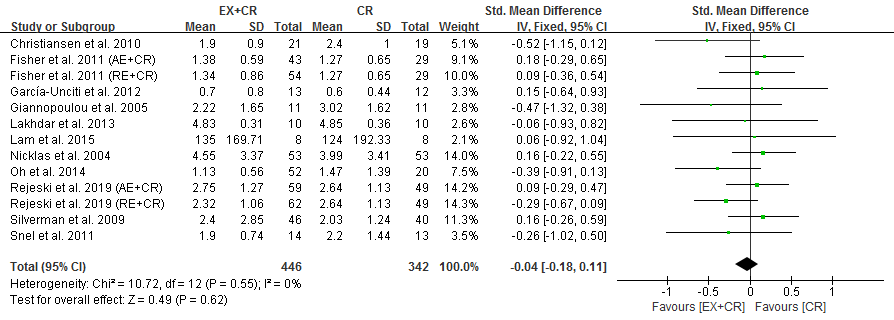
Figure 2S.** Forest plot of the effects of exercise plus calorie restriction (EX+CR) intervention and CR alone on IL-6 changes in overweight and obese adults.

**Figure 3S.** Forest plot of the effects of exercise plus calorie restriction (EX+CR) intervention and CR alone on TNF-α changes in overweight and obese adults.**
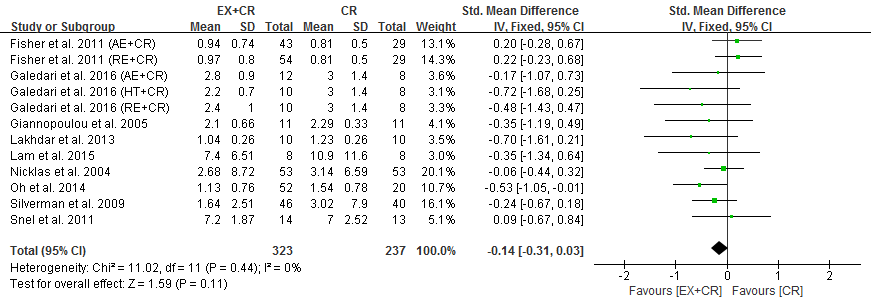
**
